# Supplementary material for: Rapid meiotic prophase chromosome movements in Arabidopsis thaliana are linked to essential reorganization at the nuclear envelope
Source: Nat Commun. 2024 Jul 16;15:5964. doi: 10.1038/s41467-024-50169-4 (PMC11252379; doi:10.1038/s41467-024-50169-4)
Supplement: Supplementary file 1 — Supplementary Information [file 41467_2024_50169_MOESM1_ESM.pdf]

Supplementary Files

Rapid meiotic prophase chromosome movements in *Arabidopsis thaliana* are linked to essential reorganization at the nuclear envelope.

Cromer *et al*.

Supplementary Fig. 1: Centromere instant speeds

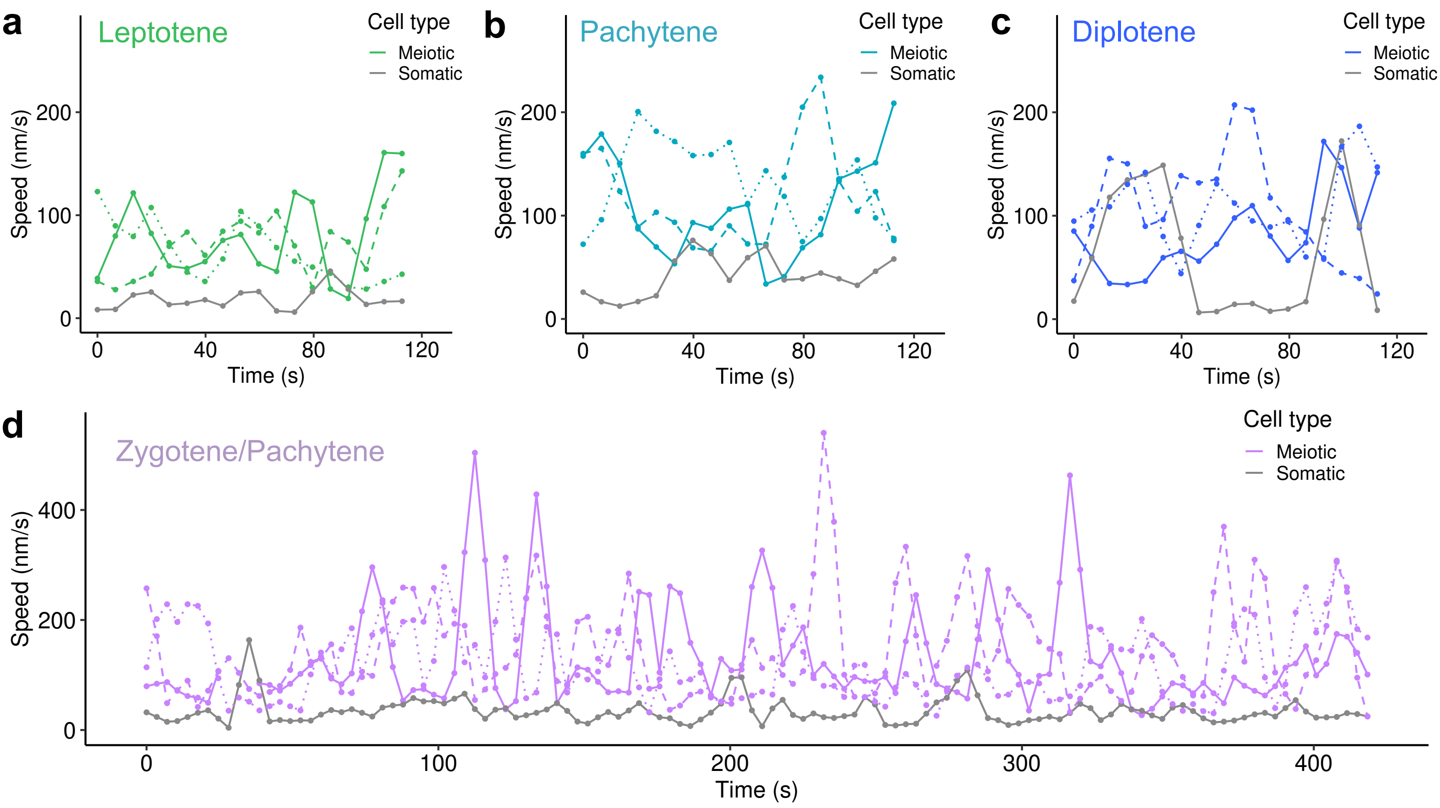

Supplementary Fig. 1: Instant speed as a function of time along individual tracks at (a) leptotene stage, (b) pachytene stage and, (c) diplotene stage. (d) Instant speed as a function of time along individual tracks for a longer acquisition at zygotene/pachytene stage. Colour curves correspond to the speed measured along different individual tracks from a same meiocyte. Different line styles (plain, dashed, dotted) are used to distinguish the different tracks. Grey curves correspond to the speed of somatic centromeres from the same anther. Source data are provided as a Source Data file.

Supplementary Fig. 2: Centromere average speeds

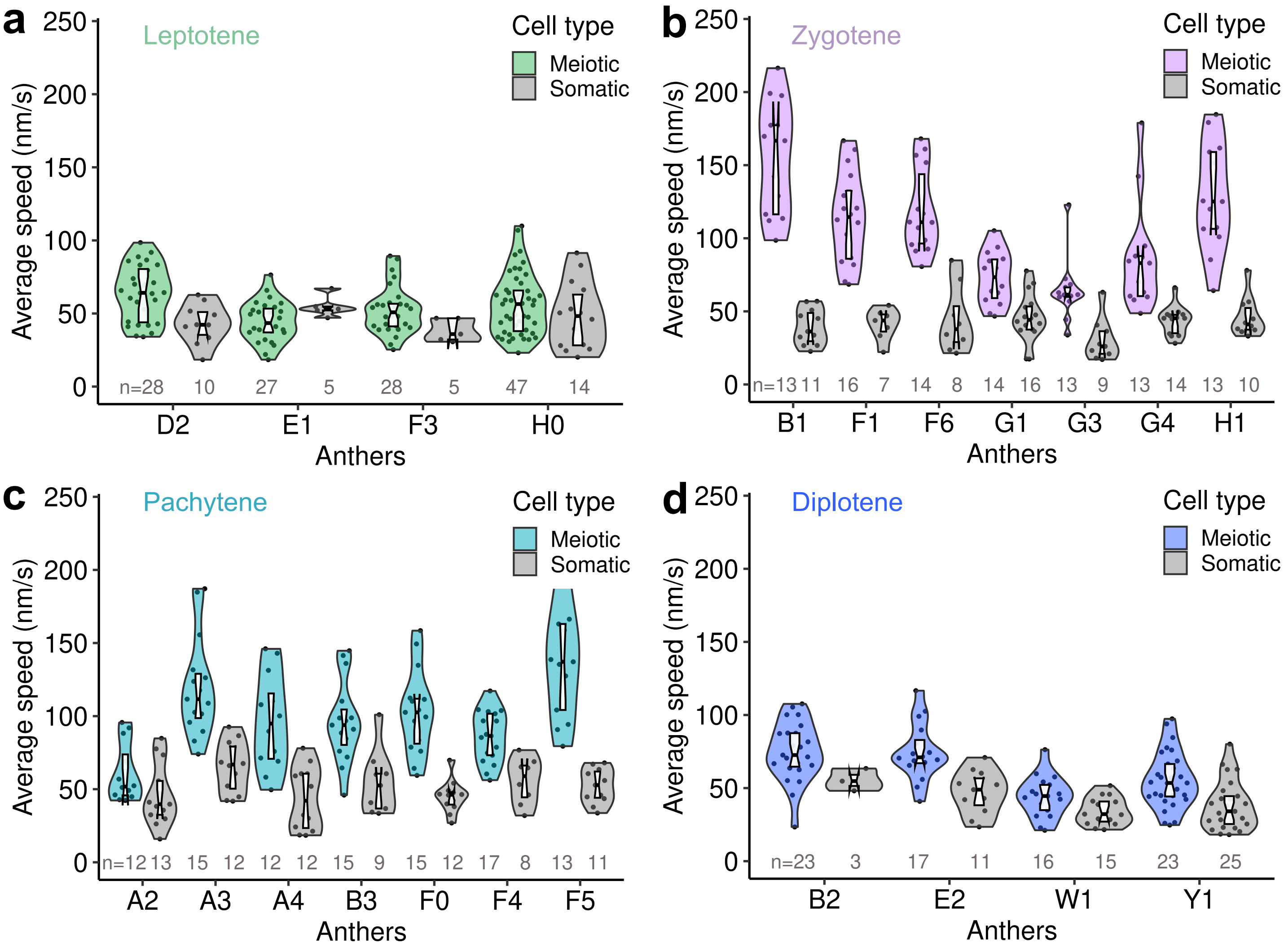

Supplementary Fig. 2: Distribution of average speeds of meiotic and somatic centromeres for the acquisitions (individual anthers) on which quantitative analyses have been performed. (a) leptotene, (b) zygotene, (c) pachytene and, (d) diplotene stages. Source data are provided as a Source Data file.

Supplementary Fig. 3: Comparison of somatic and meiotic MSDs

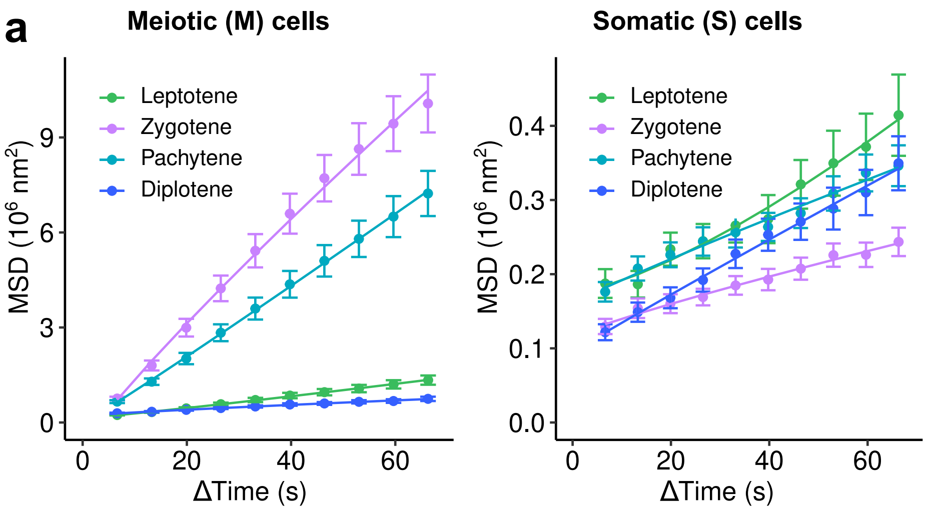

b

Stage	Cell type	α coefficient
Leptotene	M	1.1
	S	1.38
Zygotene	M	0.89
	S	0.87
Pachytene	M	1.03
	S	0.93
Diplotene	M	0.85
	S	0.97

Supplementary Fig. 3 : (a) Mean square displacement (MSD) curves for the meiotic and somatic centromeres. Error bars: average ± s.e.m.. (b) Estimated exponent α obtained by power law fit to MSD (see Materials and Methods) on meiotic (M) and somatic (S) nuclei. Source data are provided as a Source Data file.

Supplementary Fig. 4: Centromere average speeds in mutant nuclei

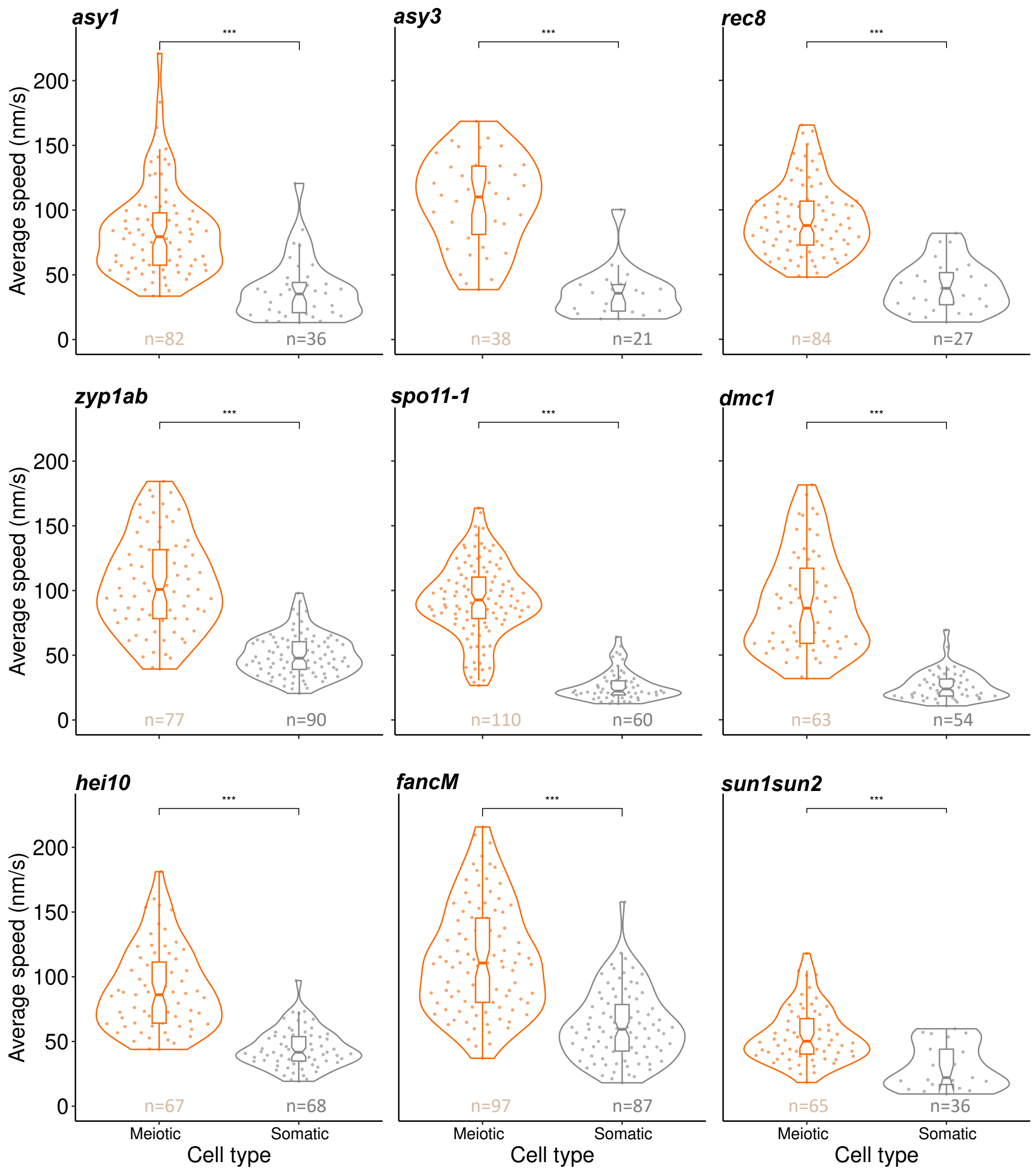

Supplementary Fig. 4 : Distribution plots of centromeric average speed in meiotic (*Orange*) and somatic (*Grey*) nuclei at zygotene-pachytene stage. Each point corresponds to an individual centromere track. Boxplots indicate median values and interquartile ranges. Horizontal brackets show the result of pairwise comparisons (mixed-effects model, with anther and cell as nested random factors, likelihood ratio test of difference between meiotic and somatic nuclei: p=2.0e-07 (*asy1*), 2.0e-05 (*asy3*), 1.9e-05 (*rec8*), 1.3e-06 (*zyp1ab*), 3.8e-10 (*spo11-1*), 2.1e-05 (*dmc1*), 1.2e-04 (*hei10*), 5.9e-05 (*fancM*), 2.2e-04 (*sun1sun2*)). Source data are provided as a Source Data file. ***: P<0.001.

Supplementary Fig. 5: Reorganisation of the NE the during meiotic prophase I

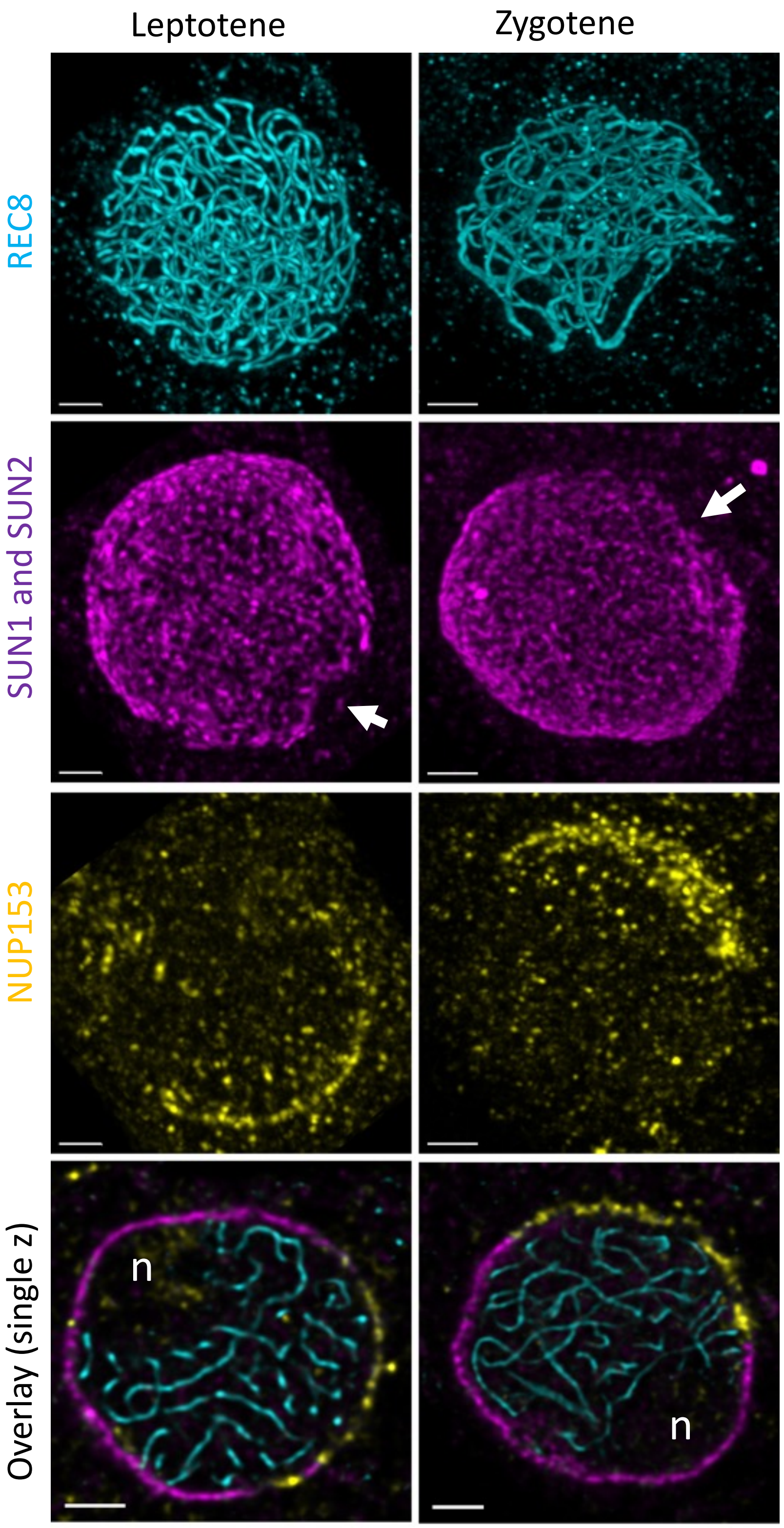

Supplementary Fig. 5: Immunolocalization of REC8 (cyan), SUN1 and SUN2 (magenta) and NUP153 (yellow) on wild-type meiocytes. Arrows indicate areas of the NE where the SUN1 and SUN2 signal is depleted and NUP153 accumulates. The first three lines correspond to maximum intensity projections of the entire z-stacks. The last line represents a single z-slice. n: nucleolus; Scale bar: 2 μm.

Supplementary Fig. 6:
SUN1 and SUN2 dynamics during male meiotic prophase

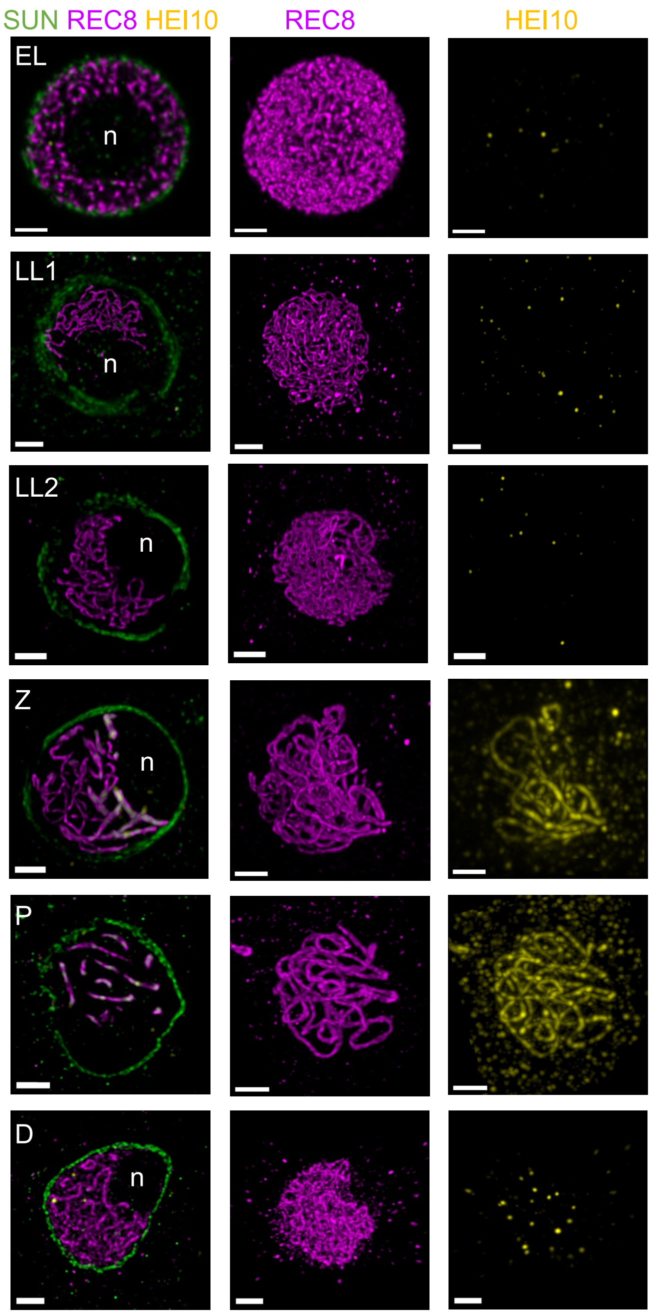

Supplementary Fig. 6: For each cell from Figure 6 (main text), the overlay between SUN, REC8 and HEI10 signals is provided for a single z-plane to visualise the position of the nucleolus (n) (first column); the second and third columns show maximum intensity projections of REC8 (magenta) or HEI10 signals (yellow), respectively. Scale bars: 2µm. EL= early leptotene, LL= late Leptotene, Z=zygotene, D= Diplotene.

Supplementary Fig. 7:Telomeres dynamics in *A. thaliana* prophase

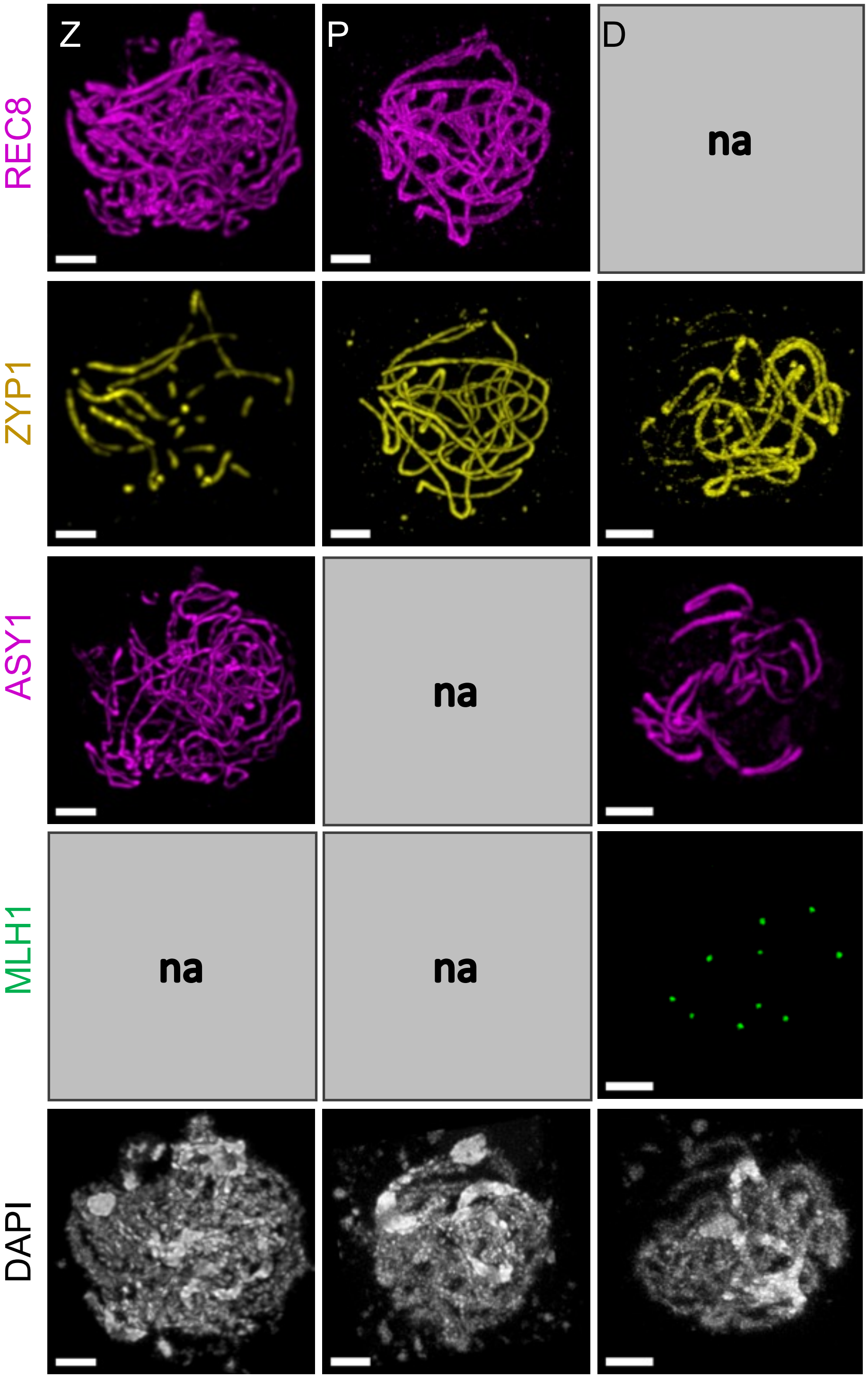

Supplementary Fig. 7: Individual channels or each cell from Figure 8 (main text). The 3D-preserved male meiocytes were DAPI-stained and immuno-labelled for different markers: REC8, ZYP1, ASY1, or MLH1. Z: Zygotene, P: Pachytene, D: Diplotene. All images are maximum intensity projections of the whole z-stack. Scale bar; 2μm.

Supplementary Fig. 8 : Identification of the NOR-containing regions

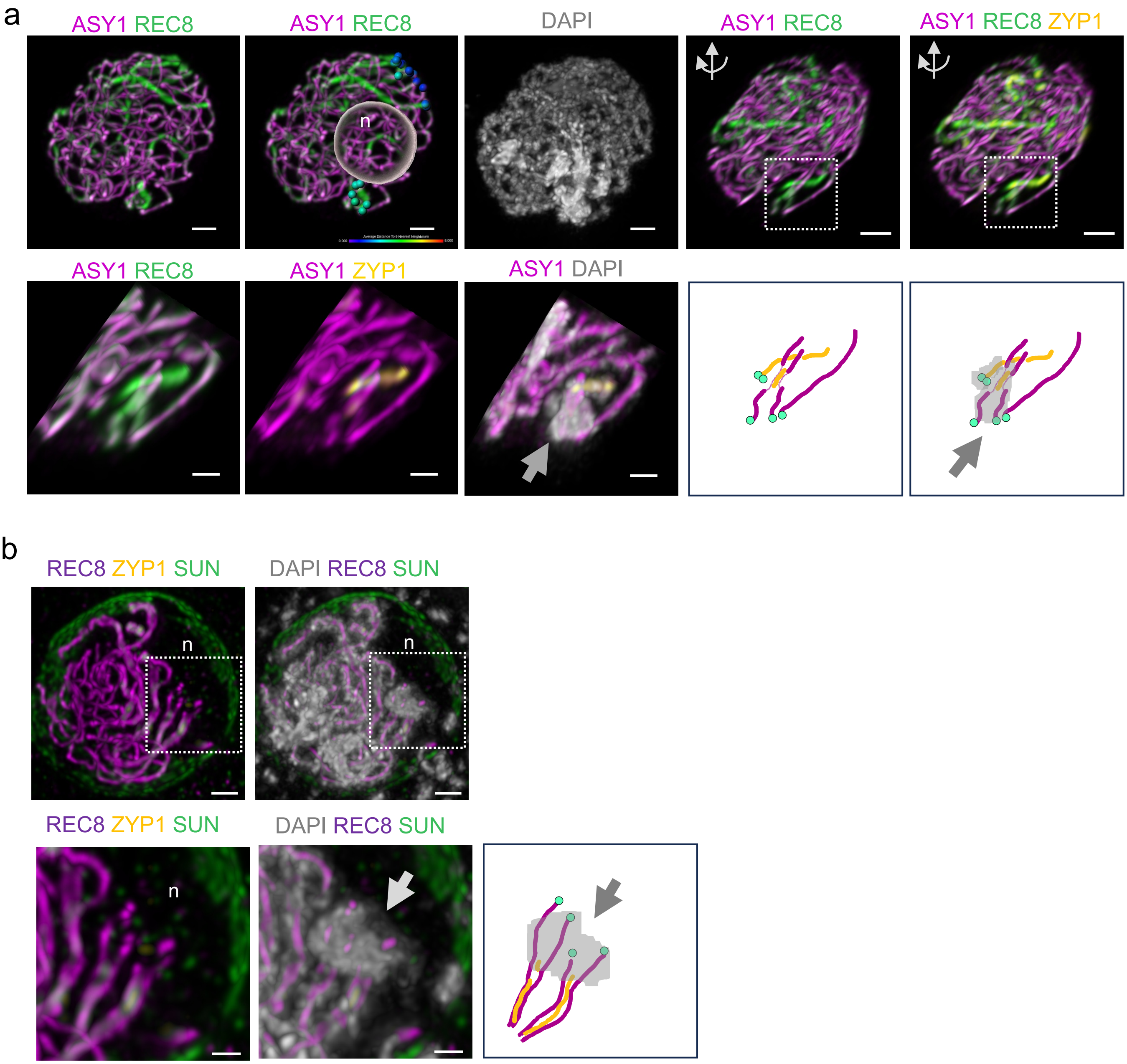

Supplementary Fig. 8: Two examples of the identification of the extremities of the short arms of chromosomes 2 and 4, which carry the NORs. These chromosome extremities are observed in close proximity to the nucleolus (n) and are embedded in the KNOB heterochromatin (grey arrows). a: A zygotene nucleus immunolabelled against ASY1 (magenta), REC8 (green) and ZYP1 (yellow). b: A zygotene nucleus immunolabelled against REC8 (magenta), SUN1 and SUN2 (SUN, green) and ZYP1 (yellow).
The first rows of a and b show general views of the cells, the second rows a zoom-in on the area indicated by a dotted square. For each cell, diagrams are provided which show a simplified representation of the signals. Telomere positions are indicated by green spots; KNOB heterochromatin by a grey diffuse area and a grey arrow.
Scale bars, general views: 2 μm. Scale bars, zoom-in, 0.7 μm. n: nucleolus.

Supplementary Fig. 9: immunostaining on *sun1 sun2.*

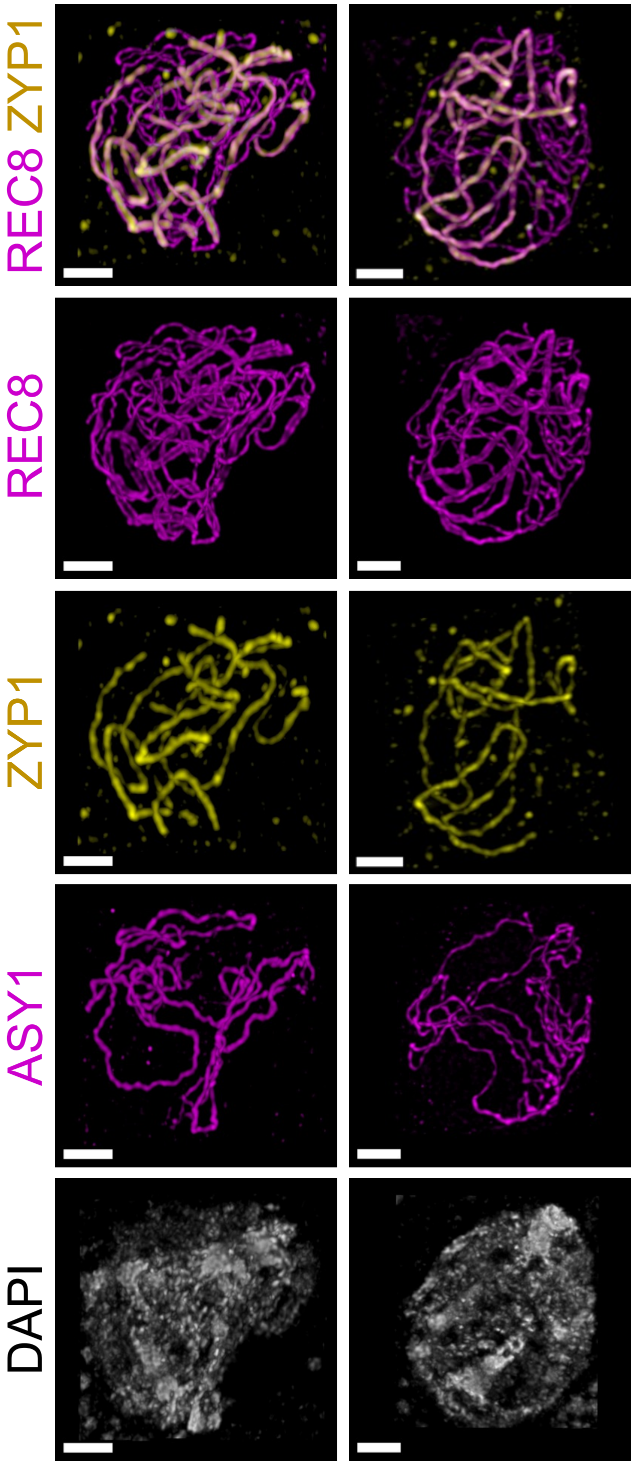

Supplementary Fig. 9: Individual fluorescent signals for each cell from Figure 9 (main text) are shown. All images are maximum intensity projections. Scale bars: 2 μm. 3D movies of the z stack are shown in Supplementary Movies 13 and 14.

Supplementary Fig. 10 : Nucleolus position and telomere dynamics in wild type

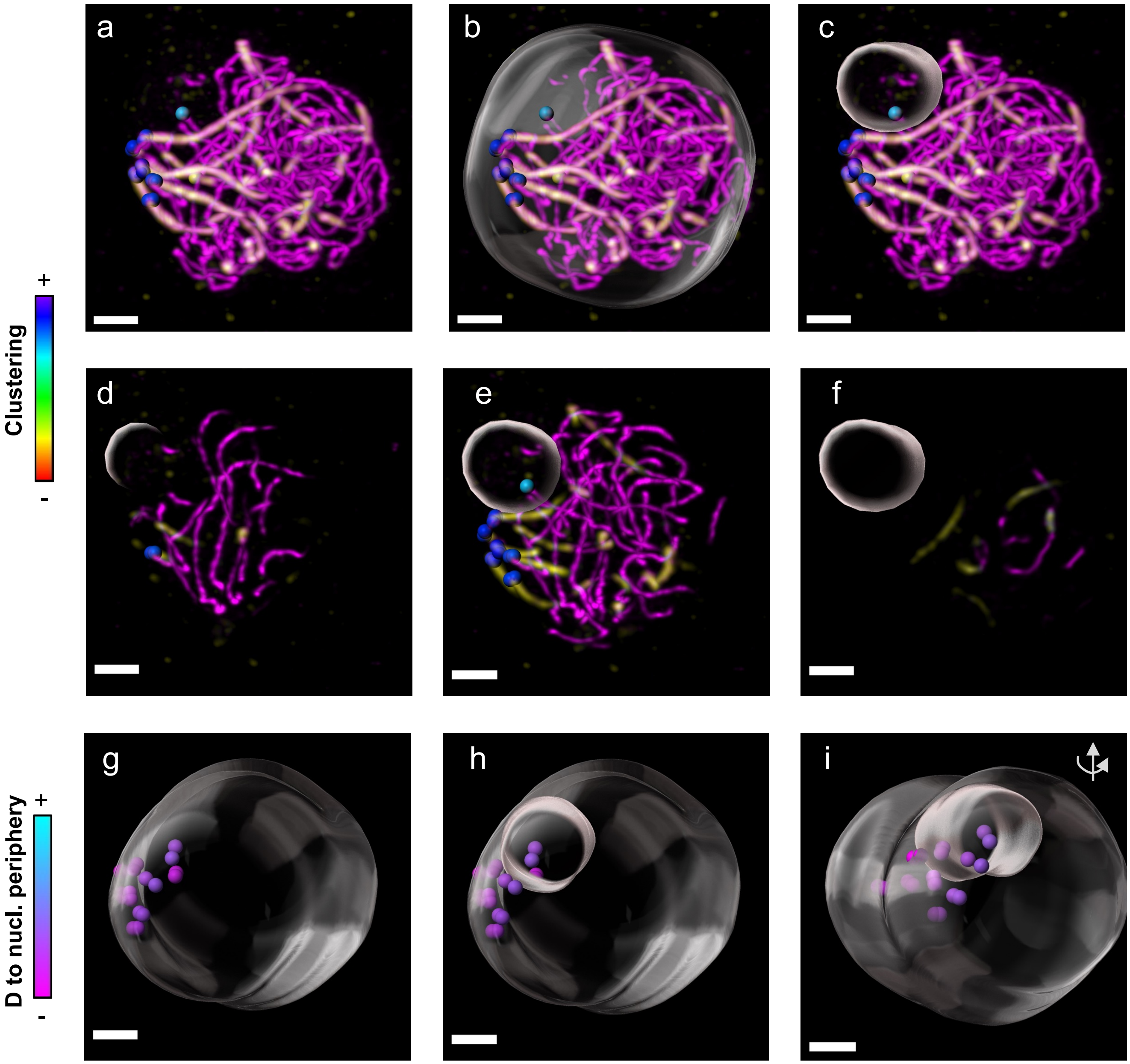

Supplementary Fig. 10: Immunostaining of a wild-type male meiocyte with anti-REC8 (magenta) and anti-ZYP1 (yellow). Chromosome extremities are indicated by coloured spots. Nuclear periphery (b, g-i) and nucleolus segmentation (c, d-f, h, i) are presented as transparent surfaces.

a-f: Maximum intensity projection of the z-stack (a-c) or of a selection of z-slices (d-f). Telomere colour code represents the intensity of the telomere clustering.

g-h: Telomeres, nuclear envelope and nucleolus segmentation seen from different angles. Telomeres dots are coloured according to their distance to the nuclear periphery. Scale bars: 2 μm.

Supplementary Fig. 11: Whole genome sequencing analysis

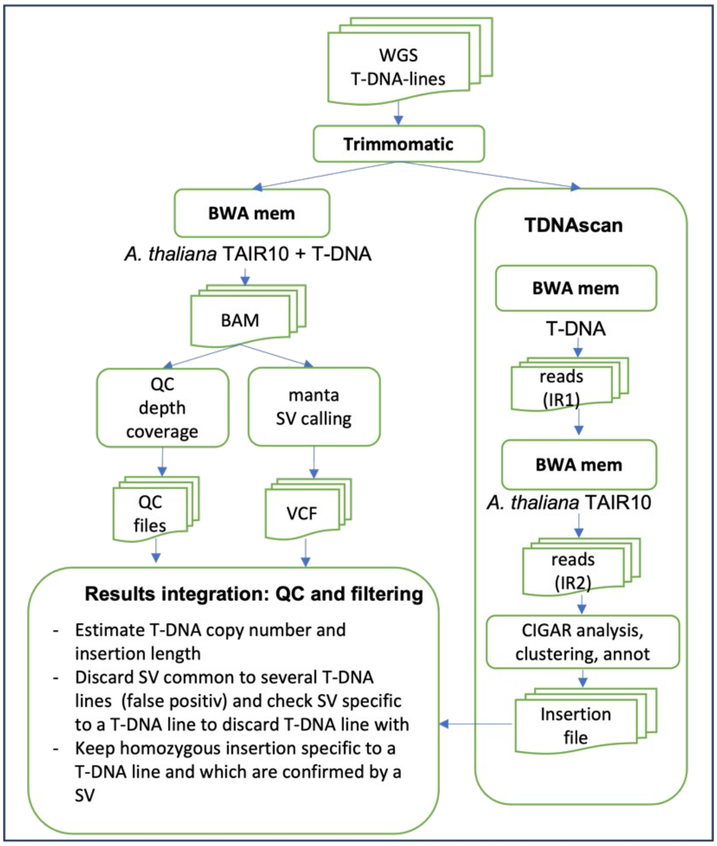

Supplementary Fig. 11: Schematic representation of the workflow set up to analyze the genomic sequences of the T-DNA reporter lines.

Supplementary Table 1:
Telomere bouquet quantification in wild-type meiocytes

stage	N (cells)	Nb of cells showing telomere bqt (%)	Average nb of telomeres in the bqt* (mean ± SD, nb of cells with Bqt)
Zygotene	14	14 (100%)	10 ± 2.3 (n=14)
Pachytene	17	15 (88%)	9.0 ± 3 (n=15)
Diplotene	15	7 (47%)	7.9 ± 1.2 (n=7)

Supplementary Table 1: This table shows the quantification of telomere bouquet formation in wild-type meiocytes at different stages. The proportion of cells of a given stage displaying a telomere bouquet is given as well as the average number of telomeres per bouquet. *: Cells with no bouquet have been excluded from this quantification. The maximum number of telomeres in a bouquet is 16 because the 4 NOR-containing arms (short arms of chromosomes 2 and 4) have been excluded from the quantification of telomeres clustering.
Bqt: “bouquet”.

Supplementary Table 2: Primer List

Primer name	Sequence (5' to 3')	use	
sun1-1U	GGGGTTATTTCAATGACAATAACCGAG	*sun1-1* genotyping	sun1-1U/sun1-1L : Wt allele
sun1-1L	GATGCGTTTTAAAGATTAACAGTATAAATTGG		sun1-1L /LbSUN1-1 : mutant allele
LbSUN1	GCCTTTTCAGAAATGGATAAATAGCCTTGCTTCC		
sun2-2U	GACTGAGTCTAGTTCACGGCC	*sun2-2* genotyping	sun2-2U/sun2-2L : Wt allele
sun2-2L	GCTGTGACAATATGCATTGAGGAGG		sun2-2L /LbSUN2-2 : mutant allele
LbSUN2	GCCAGGTGCCCACGGATAGT		
spo11-1-3U	AATCGGTGAGTCAGGTTTCAG	*spo11-1-3* genotyping	spo11-1-3U/spo11-1-3L : Wt allele
spo11-1-3L	CCATGGATGAAAGCGATTTAG		spo11-1-3L /LbSalk2 : mutant allele
asy1-4U	TCTATGTTTGTTACGCGTTAATCAG	*asy1-4* genotyping	asy1-4U/asy1-4L : Wt allele
asy1-4L	AGGTGGCTCGTAATCTGGTGGCTGC		asy1-4M /LbSalk2 : mutant allele
asy1-4M	TACAGAAGCTTCATCACTAT		
asy3-1U	AGATCTCTCATTAGTCCAATTGGC	*asy3-1* genotyping	asy3-1U/asy3-1L : Wt allele
asy3-1L	AAGACAAGGGTTCAAATGATGATC		asy3-1L /LbSalk2 : mutant allele
rec8-3U	CTCATATTCACGGTGCTCCC	*rec8-3* genotyping	rec8-3U/rec8-3L : Wt allele
rec8-3L	GGGGGAAAAGAGAAAGGTTC		rec8-3L /Lb3Sail : mutant allele
zyp1-1U	ATAGATCGATTTCGTCATCT	*zyp1-1* genotyping	zyp1-1U/zyp1-1L MboI digestion
zyp1-1L	GAGGTGAAATATGAATCTGCT		
hei10-2U	TTGTGACCATTAGGACTTCCACC	*hei10-2* genotyping	hei10-2U/hei10-2L : Wt allele
hei10-2L	GGAGAACAGAGAGACCATTCGCAAA		hei10-2L /LbSalk2 : mutant allele
dmc1-2U	TTTTTAATTGTTTACAGAGGAAATCAG	*dmc1-2* genotyping	dmc1-2U/dmc1-2L : Wt allele
dmc1-2L	TCCACTCGGAATAAAGCAATG		dmc1-2L /Lb3Sail : mutant allele
fancm-9U	ACCAAGTAGTTAGTAAGACATG	*fancm-9* genotyping	fancm-9U/fancm-9L : Wt allele
fancm-9L	GAATCATCAAGTTGTTTCCGA		fancm-9L /LbSalk2 : mutant allele
LB3Sail	TAGCATCTGAATTTCATAACCAATCTCGATACAC	*rec8-3*, *dmc1-2* genotyping	mutant allele genotyping
LbSalk2	GCTTTCTTCCCTTCCTTTCTC	*spo11-1-3, asy1-4, asy3-1, hei10-2, fancm-9* genotyping	mutant allele genotyping
GFPCENH3U	AGAAGAACGGCATCAAGGTG	GFP-CENH3 genotyping	GFPCENH3U/GFPCENH3L : reporter detection
GFPCENH3L	CTGAGAAGATGAAGCACCGGCGATAT		
REC8RFPU	GGACCAAACAGAGGAATTTGGG	REC8-RFP genotyping	REC8RFPU/REC8RFPL : reporter detection
REC8RFPL	CCTGGCTACCAGCTTCATGTAC		
SUN1GFPU	GGGGTTATTTCAATGACAATAACCGAG	SUN1-G FP genotyping	SUN1GFPU/SUN1GFPL : reporter detection
SUN1GFPL	GCGGTCACGAACTCCAGCA		
SUN2GFPU	GCTGTGACAATATGCATTGAGGAGG	SUN2-GFP genotyping	SUN2GFPU/SUN2GFP3L : reporter detection
SUN2GFPL	GCGGTCACGAACTCCAGCA		
NUP54RFPU	GAAGCGAGACATGAGGGATATGG	NUP54-RFP genotyping	NUP54RFPU/NUP54RFPL : reporter detection
NUP54RFPL	GGTGATGTCCAGCTTGATG		
CRWN1YFPU	GGGCTGACTCGGATGGTGAAG	CRWN1-YFP genotyping	CRWN1YFPU/CRWN1YFPL : reporter detection
CRWN1YFPL	GCGGTCACGAACTCCAGCA		
CRWN2YFPU	GGATACGAACGAGGATGGAG	CRWN2-YFP genotyping	CRWN2YFPU/CRWN2YFPL : reporter detection
CRWN2YFPL	GCGGTCACGAACTCCAGCA		
NUP54_GTWU	GGGGACAAGTTTGTACAAAAAAGCAGGCTGGTAATGACATAATAACTCTAAAAG	NUP54-GFP cloning	
NUP54_GTWL	GGGGACCACTTTGTACAAGAAAGCTGGGTCTGAGTCTAGTGCCATTTCTGTATC		

Supplementary Table 3: : Whole genome sequencing results

Reporter line	Ref.	Nb of insertion locus	Nb of T-DNA copies*	T-DNA position	
				Chromosome	bp (TAIR10)
**REC8-RFP**	Kuttig 2022	1	~2 (59x/[20-28x])	Chr1	27,079,816
**NUP54-RFP**	This study	1	~1 (52x/[30-42x])	Chr2	8,740,081
**GFP-CENH3**	Ravi 2010	1	~1 (10x/[16-28x])	Chr3	14,662,576

*: calculated based on the ratio between the coverage of the T-DNA and the average of the genome.

Supplementary Table 3: Summary of the results obtained out of the whole genome sequencing of the T-DNA reporter lines.

Supplementary Table 4: Time-lapse acquisition parameters - Wild type anthers

Genotype	Acquisition number	Meiotic stages[1]	markers	figure nb	image size (pixel nb)	number of Z slices per stack	Z-step size (µm)	time interval[2]	number of time frames	film duration	cumulative time per stage
Wt		Zygotene/ Pachytene*	SUN2-GFP, NUP54-RFP, GFP-CENH3	Fig. 5. SupData_7	1240 x 1240	13	1.04	31"4	20	10'28"	22'34"
Wt		Zygotene/ Pachytene*	REC8-RFP,GFP-CENH3	Fig.1. SupData_1	256 x 256	11	1.57	3"63	200	12'06"	
Wt	Lepto_D2	Leptotene[#]	REC8-RFP,GFP-CENH3	Fig.2. SupData_2	400x150	16	1.04	6"629	18	1'59"	5'57"
Wt	Lepto_E1	Leptotene[#]	REC8-RFP,GFP-CENH3	Fig.2. SupData_2	400x150	16	1.04	6"629	18	1'59"	
Wt	Lepto_F3	Leptotene[#]	REC8-RFP,GFP-CENH3	Fig.2. SupData_2	400x150	16	1.04	6"629	18	1'59"	
Wt	Zygo_B1	Zygotene[#]	REC8-RFP,GFP-CENH3	Fig.2. SupData_2	400x150	16	1.04	6"629	18	1'59"	13'53"
Wt	Zygo_F1	Zygotene[#]	REC8-RFP,GFP-CENH3	Fig.2. SupData_2	400x150	16	1.04	6"629	18	1'59"	
Wt	Zygo_F6	Zygotene[#]	REC8-RFP,GFP-CENH3	Fig.2. SupData_2	400x150	16	1.04	6"629	18	1'59"	
Wt	Zygo_G1	Zygotene[#]	REC8-RFP,GFP-CENH3	Fig.2. SupData_2	400x150	16	1.04	6"629	18	1'59"	
Wt	Zygo_G3	Zygotene[#]	REC8-RFP,GFP-CENH3	Fig.2. SupData_2	400x150	16	1.04	6"629	18	1'59"	
Wt	Zygo_G4	Zygotene[#]	REC8-RFP,GFP-CENH3	Fig.2. SupData_2	400x150	16	1.04	6"629	18	1'59"	
Wt	Zygo_H1	Zygotene[#]	REC8-RFP,GFP-CENH3	Fig.2. SupData_2	400x150	16	1.04	6"629	18	1'59"	
Wt	Pachy_A2	Pachytene[#]	REC8-RFP,GFP-CENH3	Fig.2. SupData_2	400x150	16	1.04	6"629	18	1'59"	11'54"
Wt	Pachy_A3	Pachytene[#]	REC8-RFP,GFP-CENH3	Fig.2. SupData_2	400x150	16	1.04	6"629	18	1'59"	
Wt	Pachy_A4	Pachytene[#]	REC8-RFP,GFP-CENH3	Fig.2. SupData_2	400x150	16	1.04	6"629	18	1'59"	
Wt	Pachy_B3	Pachytene[#]	REC8-RFP,GFP-CENH3	Fig.2. SupData_2	400x150	16	1.04	6"629	18	1'59"	
Wt	Pachy_F4	Pachytene[#]	REC8-RFP,GFP-CENH3	Fig.2. SupData_2	400x150	16	1.04	6"629	18	1'59"	
Wt	Pachy_F5	Pachytene[#]	REC8-RFP,GFP-CENH3	Fig.2. SupData_2	400x150	16	1.04	6"629	18	1'59"	
Wt	Diplo_B2	Diplotene[#]	REC8-RFP,GFP-CENH3	Fig.2. SupData_2	400x150	16	1.04	6"629	18	1'59"	7'56"
Wt	Diplo_E2	Diplotene[#]	REC8-RFP,GFP-CENH3	Fig.2. SupData_2	400x150	16	1.04	6"629	18	1'59"	
Wt	Diplo_W1	Diplotene[#]	REC8-RFP,GFP-CENH3	Fig.2. SupData_2	400x150	16	1.04	6"629	18	1'59"	
Wt	Diplo_ Y1	Diplotene[#]	REC8-RFP,GFP-CENH3	Fig.2. SupData_2	400x150	16	1.04	6"629	18	1'59"	

Supplementary Table 4: For each live acquisition conducted on individual anthers, the developmental stage of the meiocytes is given ([1]). Developmental stage has been determined either based on the shape of the meiocytes under bright field imaging (*), or after chromosome spreading and DAPI-staining ([#]). Brightfield imaging does not allow to discriminate between zygotene and pachytene stages, but is enough to discriminate early prophase (leptotene) from mid prophase (Zygotene/pachytene) or late prophase (diplotene).

The provided acquisition parameters include: image size in pixels, number of z slices per z-stack, the z-step size, time interval between two consecutive z-stacks ([2]), total number of frames and acquisition duration. Additionally, cumulative time per stage and per genotype are provided.

Supplementary Table 5: Time-lapse acquisition parameters - Mutant anthers

Genotype	Acquisition number	Meiotic stages[1]	markers	figure nb	image size (pixel nb)	number of Z slices per stack	Z-step size (µm)	time interval[2]	number of time frames	film duration	cumulative time per stage
sun1 sun2	Z1	Zygotene/ Pachytene*	REC8-RFP,GFP-CENH3	Fig.4	512x512	11	1.57	6"16	65	6'40"	27'24"
sun1 sun2	Z2	Zygotene/ Pachytene*	REC8-RFP,GFP-CENH3	Fig.4	512x512	14	1.57	8"67	60	8'40	
sun1 sun2	Z3	Zygotene/ Pachytene*	REC8-RFP,GFP-CENH3	Fig.4	512x512	18	1.57	7"7	47	6'01"	
sun1 sun2	Z4	Zygotene/ Pachytene*	REC8-RFP,GFP-CENH3	Fig.4	512x512	21	1.57	6"37	57	6'03"	
hei10	Z7	Zygotene/ Pachytene*	GFP-CENH3	Fig.4	400x150	16	1.04	6"62	19	2'05"	10'25"
hei10	Z8	Zygotene/ Pachytene*	GFP-CENH3	Fig.4	400x150	16	1.04	6"62	19	2'05"	
hei10	Z9	Zygotene/ Pachytene*	GFP-CENH3	Fig.4	400x150	16	1.04	6"62	19	2'05"	
hei10	Z10	Zygotene/ Pachytene*	GFP-CENH3	Fig.4	400x150	16	1.04	6"62	19	2'05"	
hei10	Z11	Zygotene/ Pachytene*	GFP-CENH3	Fig.4	400x150	16	1.04	6"62	19	2'05"	
zyp1	Z10	Zygotene/ Pachytene*	REC8-RFP,GFP-CENH3	Fig.4	400x150	16	1.04	6"62	19	2'05"	8'20"
zyp1	Z11	Zygotene/ Pachytene*	REC8-RFP,GFP-CENH3	Fig.4	400x150	16	1.04	6"62	19	2'05"	
zyp1	Z12	Zygotene/ Pachytene*	REC8-RFP,GFP-CENH3	Fig.4	400x150	16	1.04	6"62	19	2'05"	
zyp1	Z13	Zygotene/ Pachytene*	REC8-RFP,GFP-CENH3	Fig.4	400x150	16	1.04	6"62	19	2'05"	
asy1	Z1	Zygotene/ Pachytene*	GFP-CENH3	Fig.4	400x150	19	1.24	7"97	39	5'10"	29'39"
asy1	Z2	Zygotene/ Pachytene*	GFP-CENH3	Fig.4	400x150	18	1.24	7"53	40	5'01"	
asy1	Z4	Zygotene/ Pachytene*	GFP-CENH3	Fig.4	400x150	18	1.24	7"56	40	5'02"	
asy1	Z5	Zygotene/ Pachytene*	GFP-CENH3	Fig.4	400x150	18	1.24	7"53	40	5'01"	
asy1	Z6	Zygotene/ Pachytene*	GFP-CENH3	Fig.4	400x150	23	1.24	9"65	27	4'20"	
asy1	Z7	Zygotene/ Pachytene*	GFP-CENH3	Fig.4	400x150	17	1.24	7"11	43	5'05"	
asy3	Z2	Zygotene/ Pachytene*	REC8-RFP,GFP-CENH3	Fig.4	400x150	18	1.24	7"55	40	5'02"	15'00"
asy3	Z3	Zygotene/ Pachytene*	REC8-RFP,GFP-CENH3	Fig.4	400x150	17	1.24	7"13	42	4'59"	
asy3	Z5	Zygotene/ Pachytene*	REC8-RFP,GFP-CENH3	Fig.4	406x152	17	1.24	7"13	42	4'59"	
rec8	Z1	Zygotene/ Pachytene*	NUP54-RFP,GFP-CENH3	Fig.4	400x150	18	1.24	7"55	24	3'01"	16'09"
rec8	Z2	Zygotene/ Pachytene*	NUP54-RFP,GFP-CENH3	Fig.4	400x150	18	1.24	7"55	24	3'01"	
rec8	Z3	Zygotene/ Pachytene*	NUP54-RFP,GFP-CENH3	Fig.4	400x150	17	1.24	7"13	26	3'05"	
rec8	Z4	Zygotene/ Pachytene*	NUP54-RFP,GFP-CENH3	Fig.4	400x150	18	1.24	7"55	28	3'31"	
rec8	Z5	Zygotene/ Pachytene*	NUP54-RFP,GFP-CENH3	Fig.4	400x150	18	1.24	7"55	28	3'31"	
dmc1	Z2	Zygotene/ Pachytene*	REC8-RFP,GFP-CENH3	Fig.4	400x150	19	1.24	7"97	38	5'02"	20'36"
dmc1	Z4	Zygotene/ Pachytene*	REC8-RFP,GFP-CENH3	Fig.4	296x150	21	1.24	8"81	37	5'25"	
dmc1	Z6	Zygotene/ Pachytene*	REC8-RFP,GFP-CENH3	Fig.4	400x150	19	1.04	7"87	38	4'59"	
dmc1	Z7	Zygotene/ Pachytene*	REC8-RFP,GFP-CENH3	Fig.4	400x150	20	1.04	8"39	37	5'10"	
fancM	Z1	Zygotene/ Pachytene*	REC8-RFP,GFP-CENH3	Fig.4	400x150	16	1.04	6"62	19	2'05"	12'30"
fancM	Z2	Zygotene/ Pachytene*	REC8-RFP,GFP-CENH3	Fig.4	400x150	16	1.04	6"62	19	2'05"	
fancM	Z3	Zygotene/ Pachytene*	REC8-RFP,GFP-CENH3	Fig.4	400x150	16	1.04	6"62	19	2'05"	
fancM	Z4	Zygotene/ Pachytene*	REC8-RFP,GFP-CENH3	Fig.4	400x150	16	1.04	6"62	19	2'05"	
fancM	Z5	Zygotene/ Pachytene*	REC8-RFP,GFP-CENH3	Fig.4	400x150	16	1.04	6"62	19	2'05"	
fancM	Z6	Zygotene/ Pachytene*	REC8-RFP,GFP-CENH3	Fig.4	400x150	16	1.04	6"62	19	2'05"	

Supplementary Table 5: For each live acquisition conducted on individual anthers, the developmental stage of the meiocytes is given ([1]). Developmental stage has been determined either based on the shape of the meiocytes under bright field imaging (*), or after chromosome spreading and DAPI-staining ([#]). Brightfield imaging does not allow to discriminate between zygotene and pachytene stages, but is enough to discriminate early prophase (leptotene) from mid prophase (Zygotene/pachytene) or late prophase (diplotene).
The provided acquisition parameters include: image size in pixels, number of z slices per z-stack, the z-step size, time interval between two consecutive z-stacks ([2]), total number of frames and acquisition duration. Additionally, cumulative time per stage and per genotype are provided.

Supplementary Table 6: Track measures

Measure	Definition
Instantaneous speed	$V_1 = \frac{d(\mathbf{p}_1, \mathbf{p}_2)}{\Delta t}$ $V_i = \frac{d(\mathbf{p}_i, \mathbf{p}_{i-1}) + d(\mathbf{p}_i, \mathbf{p}_{i+1})}{2\Delta t}$ $V_n = \frac{d(\mathbf{p}_{n-1}, \mathbf{p}_n)}{\Delta t}$
Average speed	$\bar{V} = \frac{1}{N}\sum_i V_i$
Turning angle	$TA_i = \arccos\left(\frac{\mathbf{q}_i \cdot \mathbf{q}_{i+1}}{\lVert\mathbf{q}_i\rVert\lVert\mathbf{q}_{i+1}\rVert}\right)$ with $\mathbf{q}_i = \mathbf{p}_i - \mathbf{p}_{i-1}$ and $\mathbf{q}_{i+1} = \mathbf{p}_{i+1} - \mathbf{p}_i$
Total displacement	$TD = \sum_{i=1}^{N-1} d(\mathbf{p}_i, \mathbf{p}_{i+1})$
Maximal distance	$MD = \max_{i,j} d(\mathbf{p}_i, \mathbf{p}_j)$
Mean-squared displacement	$MSD(k\Delta t) = \frac{1}{N-k}\sum_{i=1}^{N-k} d(\mathbf{p}_i, \mathbf{p}_{i+k})^2$
Normalized outreach ratio	$OR = \frac{MD}{TD} \cdot \sqrt{(N-1)\Delta t}$
Centroid size	$CS = \sqrt{\frac{1}{N}\sum_{i=1}^{N} d(\bar{\mathbf{p}}, \mathbf{p}_i)^2}$ with $\bar{\mathbf{p}} = 1/N\sum_i \mathbf{p}_i$

Supplementary Table 6: Definitions are given for a track of N positions p*1, ….,* p*N* observed at times with constant interval Δt.

Supplementary Table 7: List of the primary antibodies

Target protein	host	dilution	references
REC8	rat	1/250	[1]
REC8	rabbit	1/250	[1]
HEI10	chicken	1/10,000	[2]
ASY1	Guinea pig	1/250	[3]
ZYP1ab	rat	1/250	[4]
MLH1	rabbit	1/200	[4]
SUN1 and SUN2	rabbit	1/100	Agrisera AS18 4224
NPC (NUP153)	mouse	1/100	Abcam ab24700 and [5]

1.Cromer, L. et al. Centromeric Cohesion Is Protected Twice at Meiosis, by SHUGOSHINs at Anaphase I and by PATRONUS at Interkinesis. Current Biology 23, 2090–2099 (2013).

2.Chelysheva, L. et al. The Arabidopsis HEI10 Is a New ZMM Protein Related to Zip3. PLoS Genet 8, e1002799 (2012).

3.Hurel, A. et al. A cytological approach to studying meiotic recombination and chromosome dynamics in Arabidopsis thaliana male meiocytes in three dimensions. Plant J 95, 385–396 (2018).

4.Higgins, J. D., Sanchez-Moran, E., Armstrong, S. J., Jones, G. H. & Franklin, F. Chris. H. The Arabidopsis synaptonemal complex protein ZYP1 is required for chromosome synapsis and normal fidelity of crossing over. Genes Dev. 19, 2488–2500 (2005).

5.Xu, X. M. et al. NUCLEAR PORE ANCHOR, the Arabidopsis Homolog of Tpr/Mlp1/Mlp2/Megator, Is Involved in mRNA Export and SUMO Homeostasis and Affects Diverse Aspects of Plant Development. The Plant Cell 19, 1537–1548 (2007).
