# Supplementary material for: Rapid meiotic prophase chromosome movements in Arabidopsis thaliana are linked to essential reorganization at the nuclear envelope
Source: Nat Commun. 2024 Jul 16;15:5964. doi: 10.1038/s41467-024-50169-4 (PMC11252379; doi:10.1038/s41467-024-50169-4)
Supplement: Supplementary file 3 — Description of Additional Supplementary Files [file 41467_2024_50169_MOESM3_ESM.pdf]

Description of Additional Supplementary file

Supplementary Movie 1:

Meiosis live imaging to study prophase chromosome movements Live imaging and centromere tracking on a wild-type anther expressing the GFP-CENH3 and the REC8-RFP fusions. Scale bar: 20 µm. The live imaging acquisition lasted 723 seconds.

Supplementary Movie 2:

Meiosis live imaging to study prophase chromosome movements A close up view of the acquisition shown in Supplementary Movie 1 illustrating the tracking of centromeres from a meiocyte (bottom left) and from somatic cells (upper right). Scale bar: 2 µm. The live imaging acquisition lasted 723 seconds.

Supplementary Movies 3-6:

Live imaging and centromere tracking on wild-type anthers Live imaging and centromere tracking on wild-type anthers expressing GFP-CENH3 (green) and REC8-RFP (magenta) fusions. The developmental stage of each anther was determined after live imaging acquisition by fixation, spreading and DAPI staining (see Materials and Methods). For each anther, tracking is shown for three meiocytes and several somatic centromeres. The duration of the live imaging acquisitions was 119 seconds for leptotene and zygotene, and 113 seconds for pachytene and diplotene. Scale bars: 10 µm.

Supplementary Movie 3: Leptotene

Supplementary Movie 4: Zygotene

Supplementary Movie 5: Pachytene

Supplementary Movie 6: Diplotene

Supplementary Movie 7:

Comparison of the mobilities of NE and centromere markers Live imaging acquisition on a wild-type meiocyte expressing the NUP54-RFP (magenta), the GFP CENH3 and SUN2-GFP fusions (green). Scale bar: 2 µm. The live imaging acquisition lasted for 597 seconds.

Supplementary Movie 8:

SUN1 and SUN2 dynamics during male meiotic prophase 3D view animation of the LL2 nucleus from Figure 6 (main text). Identified chromosome ends are marked by a grey sphere. The REC8 signal is shown in magenta, the SUN signal is shown in green, the HEI10 signal is shown in yellow. Scale bar: 3µm.

Supplementary Movie 9:

SUN1 and SUN2 dynamics during male meiotic prophase 3D view animation of the Z nucleus from Figure 6 (main text). Identified chromosome ends are marked by a grey sphere. On C, the four chromosome ends embedded in the KNOB heterochromatin are shown by a blue spheres. They correspond to the two pairs of chromosomes 2 and 4 carrying the NOR regions. The REC8 signal is shown in magenta, the SUN signal is shown in green, the HEI10 signal is shown in yellow, and the DAPI signal is shown in grey. Scale bar: 2µm.

Supplementary Movies 10-11-12

3D view animations of the cells shown in Figure 8 (main text) and Supplementary Fig. 7. (Movie 10=Zygotene cell, Movie 11=Pachytene cell, Movie 12=Diplotene cell). For each cell, the 3D reconstruction of the z-stack, the location of the telomeres (spots), and the position of the nuclear periphery (transparent sphere) are shown. Two different coloring of the telomeres are shown successively, first based on their distance to the NE and then based on the clustering intensity. The colour codes are the same as in Figure 8 (main text). Scale bars: 2µm.

Supplementary Movies 13

3D movie stack images of the cells shown in Figure 9A and Supplementary Fig. 9A. In cyan: ASY1 signal, in magenta: REC8 signal, in yellow: ZYP1 signal. Scale bars: 2 µm.

Supplementary Movies 14

3D movie stack images of the cells shown in Figure 9B and Supplementary Fig. 9B. In cyan: ASY1 signal, in magenta: REC8 signal, in yellow: ZYP1 signal. Scale bars: 2 µm.
