# Supplementary material for: Rapid meiotic prophase chromosome movements in Arabidopsis thaliana are linked to essential reorganization at the nuclear envelope
Source: Nat Commun. 2024 Jul 16;15:5964. doi: 10.1038/s41467-024-50169-4 (PMC11252379; doi:10.1038/s41467-024-50169-4)
Supplement: Supplementary file 18 — Reporting summary [file 41467_2024_50169_MOESM18_ESM.pdf]

nature portfolio

Corresponding author(s): Mathilde Grelon

Last updated by author(s): Apr 19, 2024

Reporting Summary

Nature Portfolio wishes to improve the reproducibility of the work that we publish. This form provides structure for consistency and transparency in reporting. For further information on Nature Portfolio policies, see our Editorial Policies and the Editorial Policy Checklist.

Statistics

For all statistical analyses, confirm that the following items are present in the figure legend, table legend, main text, or Methods section.

n/a	Confirmed	
☐	☒	The exact sample size (*n*) for each experimental group/condition, given as a discrete number and unit of measurement
☐	☒	A statement on whether measurements were taken from distinct samples or whether the same sample was measured repeatedly
☐	☒	The statistical test(s) used AND whether they are one- or two-sided<br>*Only common tests should be described solely by name; describe more complex techniques in the Methods section.*
☐	☒	A description of all covariates tested
☐	☒	A description of any assumptions or corrections, such as tests of normality and adjustment for multiple comparisons
☐	☒	A full description of the statistical parameters including central tendency (e.g. means) or other basic estimates (e.g. regression coefficient) AND variation (e.g. standard deviation) or associated estimates of uncertainty (e.g. confidence intervals)
☐	☒	For null hypothesis testing, the test statistic (e.g. *F*, *t*, *r*) with confidence intervals, effect sizes, degrees of freedom and *P* value noted<br>*Give P values as exact values whenever suitable.*
☒	☐	For Bayesian analysis, information on the choice of priors and Markov chain Monte Carlo settings
☐	☒	For hierarchical and complex designs, identification of the appropriate level for tests and full reporting of outcomes
☒	☐	Estimates of effect sizes (e.g. Cohen's *d*, Pearson's *r*), indicating how they were calculated

Our web collection on statistics for biologists contains articles on many of the points above.

Software and code

Policy information about availability of computer code

Data collection	No software was used to collect data
Data analysis	ZEN 2.3 (Blue edition) and LASX for image acquisition, Imaris 9.7.2 for image analysis.

For manuscripts utilizing custom algorithms or software that are central to the research but not yet described in published literature, software must be made available to editors and reviewers. We strongly encourage code deposition in a community repository (e.g. GitHub). See the Nature Portfolio guidelines for submitting code & software for further information.

Data

Policy information about availability of data

All manuscripts must include a data availability statement. This statement should provide the following information, where applicable:

- Accession codes, unique identifiers, or web links for publicly available datasets
- A description of any restrictions on data availability
- For clinical datasets or third party data, please ensure that the statement adheres to our policy

Accession codes are provided in the Methods section of the paper.Whole-genome Hi-Seq Illumina data were submitted to EMBL-EBI ArrayExpress (E-MTAB-13442).

Research involving human participants, their data, or biological material

Policy information about studies with human participants or human data. See also policy information about sex, gender (identity/presentation), and sexual orientation and race, ethnicity and racism.

Reporting on sex and gender	na
Reporting on race, ethnicity, or other socially relevant groupings	na
Population characteristics	na
Recruitment	na
Ethics oversight	na

Note that full information on the approval of the study protocol must also be provided in the manuscript.

Field-specific reporting

Please select the one below that is the best fit for your research. If you are not sure, read the appropriate sections before making your selection.

☒ Life sciences ☐ Behavioural & social sciences ☐ Ecological, evolutionary & environmental sciences

For a reference copy of the document with all sections, see nature.com/documents/nr-reporting-summary-flat.pdf

Life sciences study design

All studies must disclose on these points even when the disclosure is negative.

Sample size	Sample size was not predetermined. For cytology our goal was to achieve 15-20 high-quality cells per stage and genotype, though the precise count may fluctuate based on the complexity of sample preparation.
Data exclusions	For centromere movement analyses, zygotene/pachytene anthers where no centromere displacement was detected were not included in the quantitative analyses.
Replication	At least three independent plants were used for each experiment.
Randomization	na
Blinding	na

Reporting for specific materials, systems and methods

We require information from authors about some types of materials, experimental systems and methods used in many studies. Here, indicate whether each material, system or method listed is relevant to your study. If you are not sure if a list item applies to your research, read the appropriate section before selecting a response.

Materials & experimental systems

n/a	Involved in the study	
☐	☒	Antibodies
☒	☐	Eukaryotic cell lines
☒	☐	Palaeontology and archaeology
☐	☒	Animals and other organisms
☒	☐	Clinical data
☒	☐	Dual use research of concern
☐	☒	Plants

Methods

n/a	Involved in the study	
☒	☐	ChIP-seq
☒	☐	Flow cytometry
☒	☐	MRI-based neuroimaging

Antibodies

Antibodies used	The list of the primary antibodies used can be found in Supplementary Table 7. Secondary antibodies were conjugated with Alexa 488, Alexa 568, or Alexa 647 (Thermo Fisher).

Validation	Anti REC8 antibodies were validated in Cromer, L. et al. Current Biology 23, 2090–2099 (2013); Anti HEI10 in Chelysheva, L. et al. PLoS Genet 8, e1002799 (2012); Anti ASY1 in Hurel, A. et al. Plant J 95, 385–396 (2018); Anti ZYP1ab and anti MLH1 in Higgins, J. D., et al. Genes Dev. 19, 2488–2500 (2005); Anti NPC by Xu, X. M. et al. The Plant Cell 19, 1537–1548 (2007).

Animals and other research organisms

Policy information about studies involving animals; ARRIVE guidelines recommended for reporting animal research, and Sex and Gender in Research

Laboratory animals	na
Wild animals	na
Reporting on sex	na
Field-collected samples	na
Ethics oversight	na

Note that full information on the approval of the study protocol must also be provided in the manuscript.

Dual use research of concern

Policy information about dual use research of concern

Hazards

Could the accidental, deliberate or reckless misuse of agents or technologies generated in the work, or the application of information presented in the manuscript, pose a threat to:

No	Yes	
☒	☐	Public health
☒	☐	National security
☒	☐	Crops and/or livestock
☒	☐	Ecosystems
☒	☐	Any other significant area

Experiments of concern

Does the work involve any of these experiments of concern:

No	Yes	
☒	☐	Demonstrate how to render a vaccine ineffective
☒	☐	Confer resistance to therapeutically useful antibiotics or antiviral agents
☒	☐	Enhance the virulence of a pathogen or render a nonpathogen virulent
☒	☐	Increase transmissibility of a pathogen
☒	☐	Alter the host range of a pathogen
☒	☐	Enable evasion of diagnostic/detection modalities
☒	☐	Enable the weaponization of a biological agent or toxin
☒	☐	Any other potentially harmful combination of experiments and agents

Plants

Seed stocks

Most of the seed stocks used in this work were obtained from the NASC (https://arabidopsis.info/): sun1-1 (SAIL_84_G10), sun2-2 (FLAG_026E12), spo11-1-3 (SALK_146172), asy1-4 (SALK_046272), asy3-1 (SALK_143676), rec8-3 (SAIL_807_B08), zyp1-1, hei10-2 (SALK_014624), dmc1-2 (SAIL_170_F08), and fancm-9 (SALK_120621).Yuki Sakamoto and Sachihiro Matsunaga sent the CRWN1-YFP and CRWN2-YFP lines, Toshihisa Oda the SUN1-GFP and SUN2-GFP lines and Arp Schnittger the REC8-RFP line.

Novel plant genotypes

To generate the RFP-tagged NUP54, the NUP54 genomic fragment was amplified with NUP54_GTWU and NUP54_GTWL primers (SupData_15). The amplification covered NUP54 from 990 pb before ATG to the last pb before the stop codon. The PCR product was cloned and inserted into pDONR207 to create pENTR-NUP54. For plant transformation, the LR reaction was performed with the binary vector pGWB553116. pGWB553-NUP54 was introduced into the Agrobacterium tumefaciens strain C58C1. A. thaliana transformation was performed using the floral dip method: Agrobacterium tumefaciens was grown at 28–30 °C to saturation, centrifuged, and resuspended in a 1% sucrose 0.05% Silwet l-77 solution. This solution is used for dipping A. thaliana plants. Plants were maintained under glass overnight to increase humidity117. To select transformed plants, sterilized seeds were sown on culture medium with 25 µg/mL hygromycin B (Duchefa). T-DNA insertion sites for the REC8-GFP, GFP-CENH3 and NUP54-RFP lines were determined by whole-genome sequencing

Authentication

Authentification was done using PCR genotyping.
